# A framework for clinical utilization of robotic exoskeletons in rehabilitation

**DOI:** 10.1186/s12984-022-01083-7

**Published:** 2022-10-29

**Authors:** Kristen Hohl, Matt Giffhorn, Steven Jackson, Arun Jayaraman

**Affiliations:** 1 Shirley Ryan AbilityLab, 355 E. Erie St. , 60611 Chicago, IL USA; 2 Department of Physical Medicine and Rehabilitation, Feinberg School of Medicine, Northwestern University, 60611 Chicago, IL USA

**Keywords:** Gait, Robotics, Exoskeleton device, Technology

## Abstract

Exoskeletons are externally worn motorized devices that assist with sit-to-stand and walking in individuals with motor and functional impairments. The Food & Drug Administration (FDA) has approved several of these technologies for clinical use however, there is limited evidence to guide optimal utilization in every day clinical practice. With the diversity of technologies & equipment available, it presents a challenge for clinicians to decide which device to use, when to initiate, how to implement these technologies with different patient presentations, and when to wean off the devices. Thus, we present a clinical utilization framework specific to exoskeletons with four aims.

These aims are to assist with clinical decision making of when exoskeleton use is clinically indicated, identification of which device is most appropriate based on patient deficits and device characteristics, providing guidance on dosage parameters within a plan of care and guidance for reflection following utilization. This framework streamlines how clinicians can approach implementation through the synthesis of published evidence with appropriate clinical assessment & device selection to reflection for success and understanding of these innovative & complex technologies.

## Background

The evolution towards evidenced based practice in physical therapy has progressed over the past 25 years, however many barriers to effective translation to clinical practice persist [1]. One critical barrier is when a novel intervention or technology is introduced, there is a paucity of evidence and processes to guide clinicians on how it can be integrated into their everyday clinical practice.

In the current manuscript, we will discuss the clinical use of robotic exoskeletons, which have come into commercial availability since 2011. In the context of this manuscript, exoskeletons are defined as externally worn devices that assist with sit-to-stand and gait training in individuals with motor and functional impairments. They have tremendous potential to assist in the delivery of rehabilitative care through improved efficiency, decreased cost with ability to achieve a high stepping dosage and intensity, and decreased therapist-burden and risk of injury compared to other gait training strategies [2, 3]. The field of robotic technologies is rapidly evolving, with a projected growth of 26% over the next 5 years [4]. Exoskeletons currently approved for clinical use by the US FDA include Rewalk^TM,^ Ekso™, Indego™, Hybrid Assistive Limb (HAL) ^TM^ for medical use (lower limb type), Rewalk Restore™, B-Temia Keeogo + ™ and Honda Walking Assist Device (WAD)^TM^.^(5, 6)^ Table 1 describes the FDA-approved device features including level of assistance, resistance modulation, joint control, type of feedback, and stepping actuation. Exoskeletons currently are not considered standard of care in rehabilitation, however patients often seek facilities offering these advanced technologies. Given the emerging evidence of clinical utility, patient interest, and anticipated growth of the field, it is critically important clinicians can effectively evaluate and implement the use of these devices.

**Table 1:** Comparison of FDA Approved Exoskeleton Devices. Summary of current devices in marketplace with difference in joints controlled, location and type of support provided, resistance or assistance capabilities, method of stepping actuation, and minimum walking function required

	Joints Powered	Level of Support	Assistance	Resistance	Feedback	Stepping Actuation
Rewalk	Hip and Knee	Full Trunk, B LE, stance and swing	FP	No	None	Watch + Trunk Motion
Ekso	Hip and Knee	Full Trunk, B LE, stance and swing	FP to PA	Yes, swing phase	Auditory, visual tactile, spatiotemporal	Therapist trigger, weight shift, or free stepping
Indego	Hip and Knee	Lower lumbar, B LE, stance and swing	FP to PA	No	Auditory, Tactile, spatiotemporal	Forward trunk lean or free stepping
HAL for medical use	Hip and Knee	Lumbar, B LE, stance and swing	FP to PA	No	Auditory, visual, tactile, Spatiotemporal	Through bioelectric surface EMG on leg muscles
Honda WAD	Hip	B LE, swing	PA – user must be ambulatory	Yes	None	Detects and assists in free stepping
B-Temia Keeogo+	Knee	B LE, stance and swing	PA – user must be ambulatory	No	None	Detects and assists in free stepping
Rewalk Restore	Ankle	U LE, swing and propulsion	PA – user must be ambulatory	No	Visual, Spatiotemporal	Detects and assists in free stepping

B LE = bilateral lower extremity device, U LE = unilateral lower extremity device, FP = fully powered; device provides majority of power at joints and user needs little to no volitional strength to utilize; PA = partially assistive; device provides customized partial assistance to augment deficits to improve gait.

Depending on the rehabilitation facility, clinicians may have access to only one of these devices while others may have multiple options. Regardless of the device availability, practitioners must systematically assess the technology’s features related to their patient’s impairments and functional level to determine if utilization is indicated. Table2 describes the outcomes from randomized control trials to date that have focused on use of FDA approved devices compared to conventional care.

**Table 2:** Clinical outcomes for trials including diagnoses approved by FDA. Summary of objective outcome measures pre to post intervention from clinical trials investigating FDA approved diagnoses

	Device	10m Walk Test SSV or FV (meters/second)	6min Walk Test (meters)	Timed Up & Go (seconds)	Berg Balance Scale (range 0–56 points)	Lower Extremity Motor Score (range 0–50)
Sub-Acute Stroke	HAL single leg(7)	-	(2MWT) 4-20.75	-	8–22	-
	HAL single leg(8)	0.56–0.85 (FV)	92.4-156.7	33.9–16.7	-	-
	Ekso(9)	0.25–0.50**	48.60–139.24	-	-	-
Chronic Stroke	Ekso(10)	0.5–1.2**	-	33 − 24**	-	-
	Honda WAD(11)	0.7–0.94	257–375	-	43.5–48.5*	-
Sub-Acute SCI	Ekso & Ekso GT(12)	0.26–0.32	-	38.3–31.3	25.4–31.5	19.4–24.0
Chronic SCI	Ekso & Ekso GT(12)	0.30–0.37	-	35.0-27.2	25.0-28.9	14.5–14.7
	Ekso GT(13)	0.17–0.22	50–67	71 − 55	-	-

SSV = Self-selected velocity, FV = fast-velocity, 2MWT = 2min walk test. * estimated from % change and graphs; ** approximate values from graphical data only. If using these devices in an alternative frequency or dosage than used in these studies, outcomes may differ

Specifically, this manuscript focuses on diagnoses approved for use by the FDA. Studies which have investigated the sub-acute and chronic stroke populations included persons with single or unilateral stroke, with a majority including individuals greater than 55 years of age [7–11, 14, 15]. In the incomplete spinal cord injury (SCI) population, most investigations are single group interventional studies or pilot randomized trials. These studies mostly focus on inclusion of participants with incomplete (AIS C or D) injuries with upper motor neuron signs and sufficient upper extremity strength to use an assistive device. Studies focusing on participants with cervical level injuries, AIS A and B injury classification, and lower motor neuron injuries are limited and with varying sample sizes of 9–52 subjects [12, 13]. It should be noted, the aim in many of these studies was to obtain FDA approval with a primary focus on establishing safety with one primary efficacy outcome. Thus in many cases, the true functionality and clinical effectiveness of these devices has not been investigated. Furthermore, these studies also do not focus on dosing, progression strategies, or rehabilitation principles critical to therapeutic intervention [16–18].

Adding more uncertainty to application of the available literature, the clinical practice guideline (CPG) for improving walking function in chronic neurological diagnoses, recommended against utilizing robotic interventions [17]. Ten of the eleven studies referenced were not the FDA approved devices focused on in this current manuscript, and eight of the studies focused on treadmill-based robots, specifically the Lokomat [17] These conclusions should be taken with caution, given the substantial differences in functionality and physical demand between the treadmill-based robots and the overground exoskeletons of current focus. Thus, understanding the current literature along with synthesis of knowledge from clinical experience regularly utilizing exoskeletons in practice was critical in developing this framework.

In this four-step framework, we focus specifically on clinical application, rather than exoskeleton use for personal mobility. As authors, we are in a unique position to propose a comprehensive framework to assist in this systematic evaluation due to having extensive experience utilizing a wide array of these exoskeletons during the research and development phase, FDA clinical trials, as well as extensive use in everyday clinical practice [2, 11, 14, 19, 20].

## Framework

**Fig. 1:**
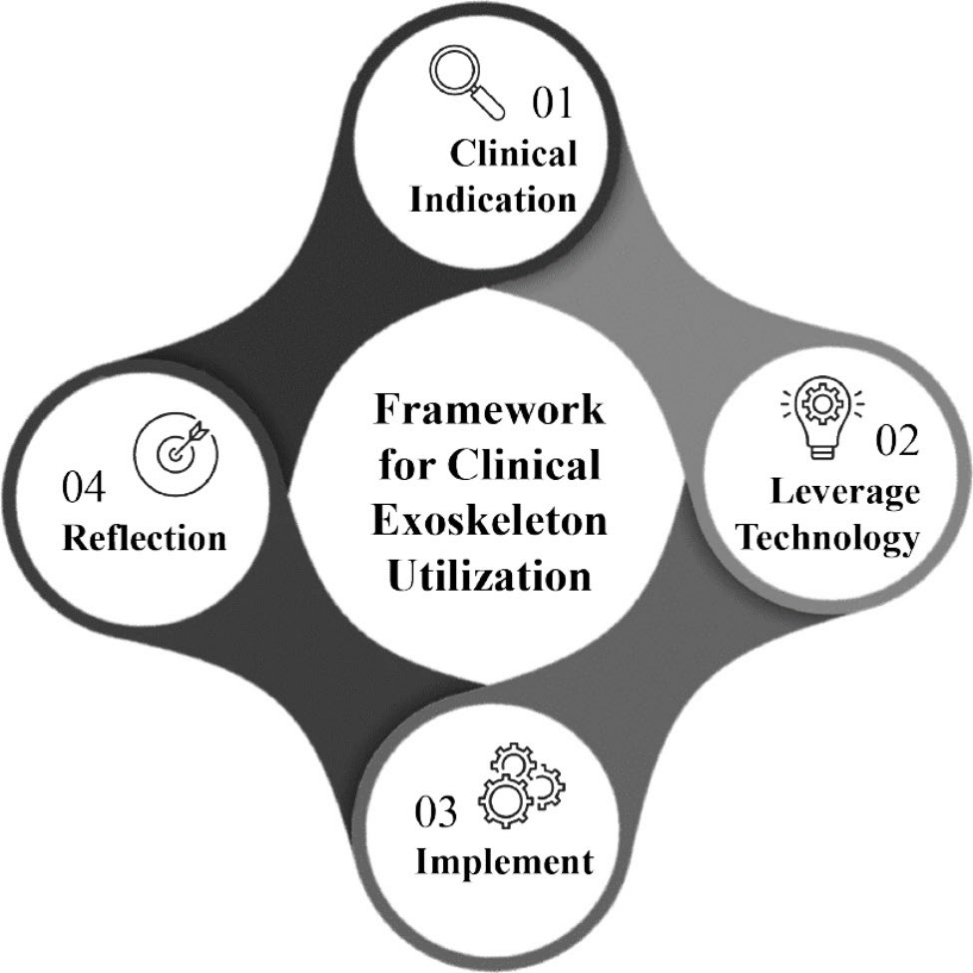
4-step clinical exoskeleton framework. Framework structures clinical decision making surrounding appropriate patient identification, leveraging suitable technology to match patient needs, implementing into a plan of care and clinical reflection to guide further use

## Step 1: Clinical indications for exoskeleton use

Clinicians performing evaluations may identify a patient is suitable for exoskeleton utilization at the beginning of an episode of care, or when challenges arise during gait training within a conventional plan of care. Often a patient’s clinical presentation does not match the exact inclusion/exclusion criteria described in the published literature. This should not preclude a clinician from considering incorporation of exoskeleton technology in the plan of care. Inclusion criteria can serve as a baseline for understanding which patient populations and presentations have been investigated to date. Because technology and software development often outpace scientific research, frequently the device investigated is an older version with fewer features or modes than what is available currently.

### Supports and unweighting

If a patient requires significant use of body weight or mobility support/aides including harnesses, bracing, strapping, and padding to be successful when gait training, clinicians may consider an exoskeleton to replace many of these supports. Multiple supports may be inefficient due to extensive initial setup time and the need for frequent in session adjustments. To promote improved stepping ability, it is recommended that a patient is unloaded no more than 30–40% of their body weight [21, 22]. If a patient requires more unweighting than this to achieve successful stepping, it may be appropriate to consider an exoskeleton which can provide almost full body weight loading through the lower extremities. Occasionally, even with positive stepping occurring, there can be considerable compensations in the frontal and sagittal planes leading to increased pain or concern for joint health. Although evidence indicates perfect kinematics are not needed for gait recovery, excessive compensations should alert clinicians to consider alternative setups and equipment available [23]. Exoskeletons, particularly those that are two jointed, can provide support and structure to minimize some of these compensations, particularly deviations that occur outside of the sagittal plane.

Looking at gait deviations occurring during each of the biomechanical subcomponents of gait during conventional intervention is another mechanism to determine if a patient may benefit from exoskeleton utilization [24]. For example, during stance stability, if a patient presents with knee collapse during stance phase or an inability to achieve hip extension in terminal stance despite unloading or therapist assistance, it may be appropriate to consider powered assistance from an exoskeleton. Conversely, for limb advancement, if a patient has limited swing phase advancement and requires two person assist to perform a positive step length, it may be appropriate to use an exoskeleton as the personnel demands are equalized between intervention approaches. Assistance from the exoskeleton motors in these instances would likely be most suited to the specific joint where the therapist identifies the primary limiter to the biomechanical subcomponent of gait is occurring. By using an exoskeleton in these instances, it allows for achievement of these biomechanically important positions to gait recovery in a more efficient manner [25, 26].

### Burden on clinician

Moreover, there can be a substantial physical burden on clinicians due to awkward crouched postures with concurrent repetitive loading while providing manual assistance during treadmill and overground gait training. Relatively high injury rates have been reported of the lower back, shoulder, and hand with tasks of repositioning in clinicians with assistance during gait training [27]. When an exoskeleton is utilized, the clinician provides assistance and stabilization in a better ergonomic position either laterally or posteriorly while in standing. This posture and body position results in significantly less strain and physical demand compared to manually assisting limbs described previously. Thus, if clinicians are reporting substantial physical burden due to the level of physical assistance a patient requires for gait training, it may be safest for the clinician to consider an exoskeleton.

## Step 2: Leveraging technology

Pairing a patient’s clinical presentation to the device design and functionality is the most critical step in effective exoskeleton utilization. A good match can result in significant benefit to the patient, while an improper pairing will likely result in lack of positive results. To effectively match a patient’s presentation to the appropriate device, clinicians must have a strong understanding of each available exoskeleton and its properties. Features such as software programs, customization of fit, stepping and support characteristics previously described in Table1 as well as progression strategies must be well understood. Four primary areas should be considered when choosing a device, which will be discussed in more detail in the following sections.

### Level of support

In choosing an exoskeleton, clinicians should start by matching the support offered by the device to the patient’s clinical presentation and specific areas of deficits. Primary considerations include where the device provides support (trunk, unilateral or B LE), which joints are assisted, and how the support can be changed. If the patient demonstrates bilateral deficits in stance phase (knee buckling/inability to achieve knee extension) even with body weight support provided, devices that provide bilateral stance phase assistance at the knee is a minimum requirement. Devices which satisfy this requirement are Rewalk, Indego, Ekso, and HAL. To narrow down options, considerations of the patient’s trunk control is appropriate. Impaired trunk control would lead to use of a device which provides full trunk support (Rewalk or Ekso) while with minor trunk control deficits, the other two devices may still accomplish the goals. Device selection would also be driven by the patient’s anticipated prognosis and thus the expected reduction in powered support required as the patient recovers. Some devices (Ekso/Indego/HAL) can provide varied amount of support from fully powered to partially powered in the legs, which is a good choice if you are anticipating improvement in patient functioning while Rewalk is unable to reduce motor support significantly.

### Pattern of assistance/ resistance

The device software programs allow for greater customization of the assistance or resistance delivered. The amount of overall motor assistance, direction of assistance (i.e. forwards or backwards), timing of delivery, as well as individualizing to specific joints or phases of gait are important considerations. Understanding what type of assistance or resistance your patient needs based on biomechanical subcomponents of gait identified in Step 1 and pairing this to the most appropriate technology and software setup, is critical to effective utilization. Devices such as Ekso, Indego and HAL can provide fully powered to partially powered assistance are likely going to encompass a great majority of clinical presentations. Uniquely, Ekso can provide resistance additionally through swing phase, which is a beneficial progression strategy for patients with limb control issues, goals to improve strength/endurance or overall progressing to reduced assistance needs. Also unique to Ekso, ambulation can be performed in the backwards and lateral direction, adding the multidirectional component important for gait recovery [18]. A patient who presents with limb swing deficits may benefit from the use of Indego’s Therapy + software or HAL, which allows for customized joint specific assistance. If decreased swing phase clearance is primarily resulting from decreased knee flexion, specific assistance can be targeted at that joint and phase of the gait cycle with the use of either of these devices or if isolated to an ambulatory individual, Keoogo + may be an option. In contrast, Ekso provides a global swing phase assistance, and is unable to target to specific joint deficits in swing phase. Devices acting at a single joint are Honda WAD, B-Temia Keeogo + and Rewalk Restore assist at the hip, knee and ankle respectively. Selection of the Honda WAD may be driven by deficits of decreased swing phase clearance, decreased step length or overall gait speed. Keeogo + may selected when there is decreased swing phase clearance due to reduce knee flexion, stance phase support deficits or other mobility limitations noted on stairs or sit to stands, primarily due to weakness or pain around the knee joint. Rewalk Restore acts unilaterally at the ankle and is more specific to swing phase deficits or propulsion deficits in a patient with unilateral deficits. This may be an appropriate selection when transitioning from an ankle foot orthosis (AFO) for decreased support/strengthening of the lower limb muscles, facilitating propulsive mechanics, increasing gait speed or step length.

### Augmented feedback

One primary benefit to utilizing specialized exoskeleton technology is that they can provide specific and valuable feedback to the patient and clinician not possible with traditional strategies. This can include spatiotemporal feedback, the amount of motor assistance required for successful stepping, auditory, tactile, or visual cues. For example, a patient with an asymmetrical gait pattern may be well suited to utilize Indego that has the capability to provide customized step length target and feedback, via an auditory cue. Vibration can be used for sagittal plane weight shifting feedback with Indego’s Motion + program. HAL uniquely provides muscle activation biofeedback via auditory and visual feedback during gait and has the capability to provide visual feedback regarding balance position. Auditory cues for weight shifting during gait as well as auditory feedback can be provided when swing motor assistance increases beyond the set level in Ekso. Both can be helpful to improve awareness, work on improving balance and consistency of stepping as motors may be reduced. Real time feedback of balance position can be provided by HAL or Ekso, which may be beneficial for a lower level patient. Spatiotemporal feedback can be provided by Ekso and Rewalk Restore, which may be more beneficial to clinicians wanting to address common deficits including step length, stance time and swing time. Summary statistics, of spatiotemporal feedback or overall stepping summary, provided by all devices, can be useful for objective feedback if device settings are changed (i.e. decreased assistance) and resulting impact on patient performance. These data can guide future decision-making regarding settings.

These examples demonstrate how these technologies can objectively provide feedback in ways not possible with traditional gait training. The learning curve of the technology can take a few sessions for patients and thus it may be advisable to provide consistent verbal feedback or cues through the technologies for desired performance early on to minimize patient frustration. Over time to facilitate carryover and motor learning, it may be beneficial to taper frequency of the previously mentioned augmented feedback. Perhaps the means remove the cues/feedback entirely, or decreasing the frequency of showing the feedback to a faded schedule and transitioning to providing knowledge of results to facilitate ongoing motor learning [28].

### Minimum function required

Lastly, all devices have a minimum functioning required for successful and safe operation. This may include strength in a particular muscle group or a minimum walking speed for the device to adequately detect walking and provide assistance at the appropriate time. Understanding if a patient meets these necessary thresholds is essential prior to integration within a plan of care. Indego therapy + requires at least 3 + hip extension strength as active assistance for hip extension is not provided in this software mode and HAL in voluntary control requires volitional activation due to its use of EMG feedback for stepping activation. Devices that require ambulatory function (Honda WAD/Rewalk Restore/B-Temia Keeogo+), utilize algorithms for detecting and assisting gait. Patients who ambulate slowly ( ~ < 0.2m/s) or have a step to gait pattern are unlikely to receive appropriate assistance from these devices and thus may be unsafe for use.

Proper decision making to select the most applicable technology related to the patient’s needs requires a strong understanding of each device. Considerations such as programmability and advanced clinical reasoning to structure and progress the training in an individualized manner is required. Inappropriate pairing of an exoskeleton and a patient’s impairment could lead to patient harm. Employing a one-size fits all approach to device and program utilization decreases the probability that the technology will effectively contribute to progression towards established patient goals.

## Step 3: Implementation

Once a particular exoskeleton is identified, clinicians must then determine an appropriate strategy for implementation. Dosage parameters such as frequency, duration, and intensity should be considered in the context of the exoskeleton use as well as how it fits in with other interventions as well as current best practice recommendations for locomotor recovery [17]. In the inpatient rehabilitation setting, medically stable is a minimum requirement to initiate treatment, including adequate spinal and cardiovascular stability for gait training. While blood pressure regulation can be a challenge acutely post injury, exoskeletons have been utilized with minimal incidences of adverse events in the inpatient rehabilitation setting [29, 30]. One potential explanation may be that exoskeletons are typically set up and donned in sitting, which allows initiation of stepping immediately upon standing and symmetrical stepping allows for blood pressure regulation. Contrasted with other intervention setups such as the Lokomat™, which require prolonged static standing leading to hypotensive events.

Table3 outlines the variability in the literature for frequency and duration of use where improvement in gait function was the primary goal. Previous studies report frequencies from 3 to 5 times a week with each session ranging from 20 to 60min of walking time, for a total of 12–40 sessions [7, 8, 10–13, 15].

**Table 3:** Components of Exoskeleton Utilization. Frequency, dosage and intensity of exoskeleton training in various FDA approved-populations studied where gait outcomes were primary focus

	Frequency and Dosage	Intensity	Total Sessions
Sub-Acute Stroke	4x/week for 4 weeks, 60min walking (n = 17) (7)	*	16
	3x/week for 4 weeks, 20min walking (n = 12) (8)	*; adjusted based on speed, support and distance	12
	5x/week for 3 weeks, 60min + 120 conventional (PT/SLP/OT) (n = 38)(9)	Defined as steps/session	15
Chronic Stroke	5x/week for 8 weeks 45min + 60min conventional (n = 20) (10)	*	40
	3x/week for 6 weeks, 45min (n = 25)(11)	RPE 12–16	18
	5x/week for 4 weeks for 45min + 60min conventional PT and OT (15)	*; Termination of session if too fatigued	20
Sub-Acute SCI	3x/week for 8 weeks, “add on” to existing therapy; up time mean 30–35min (n = 25) (12)	RPE 11–13	24
Chronic SCI	3x/week for 8 weeks, “add on” to existing therapy; up time mean 30–35min (n = 27) (12)	RPE 11–13	24
	5x/week for 3 weeks for 60min (n = 4) (13)	*	15

RPE = rating of perceived exertion; PT = physical therapy, OT = occupational therapy, SLP = speech therapy *= study did not report on how intensity was measured during session

The feasibility of implementation of these parameters may be a challenge due to insurance limitations, session duration, and other patient goal areas. Initial device sizing and setup can be time intensive however, clinicians experienced with use of a particular device can setup a patient within a similar time demand required for complex treadmill setups. Given the strong evidence for overground and treadmill gait training, it would be advisable to integrate these modes of gait training into the plan of care, even if exoskeleton training was introduced due to difficulties encountered with these modes previously. This integration of multiple modes of gait training would allow for variability in task practice as well as carryover outside of the device. Treatment sessions for each patient may be different throughout the plan of care depending on progress however, if setup takes more than ten minutes of an hour session, it may be most time efficient to focus on the exoskeleton intervention for the entire session. Separate sessions can then exclusively focus on treadmill and or overground training. Frequency of exoskeleton sessions are most helpful if performed more than once per week for learning and consistency however, execution of this is dependent on the factors noted previously. As the patient progresses to a higher level, setup typically becomes less complex and quicker to don thus affording more time for multiple gait interventions in a session. One may start with exoskeleton training and then progress to overground training within a session to facilitate carryover and motor learning [31].

## Step 4: Reflection

The final step of this framework is reflection. Reflection requires a self-assessment of what occurred, the importance of it, and any changes that are needed as a result. This step in the framework should occur following each session and may be needed during a session. Immediate problem solving and quick critical evaluation within session may be necessary in the case of poor performance, particularly if patient safety is at risk. Perhaps the patient may need more assistance, or the device setup needs modification. Often with exoskeleton use, there is a significant learning curve for the patient, requiring patience from the clinician to not immediately discount the technology, further adjust settings, or terminate prematurely. Following each session, consideration of the overall success or shortcomings should be analyzed. This should include a breakdown of potential contributors of patient factors, therapist influences, and technology fit. Some questions to consider by category are listed in Table4. Assessment of exoskeleton treatment effectiveness is dependent on patient’s goal areas and deficits. Examples may include overall decreased therapist assistance, increased stepping tolerance, decreased motor assistance required, improved step length, and/or decreased stride asymmetry etc.

Ultimately, the formal reassessment of outcomes can serve as an objective reflection of the exoskeleton intervention. Exoskeletons are a tool to accomplish the patient’s goals of improved walking function. With this focus, the goal is not to have patients take home an exoskeleton for ambulation. Thus, performing the battery of outcomes outside of the exoskeleton as recommended by the outcome measures CPG is an excellent assessment if continuing utilization is appropriate for a patient [32].

**Table 4:** Reflective questions with three foci. Reflective questions following each session directed at the patient, therapist and the technology. It is critically important to evaluate all domains to effectively assess and progress utilization

Patient	Therapist	Technology
Was the session successful in achieving goals for the session?	Can you identify any gaps in your knowledge which are needed for using this technology with this patient’s current presentation?	Is this technology still a good match for the patient’s deficits and goals?
Were there any medical factors contributing to the session goals or completion of it?	Do you need to refer back to the manual or reach out to someone (mentor/manufacturer) for guidance?	Should you try a different setup or software program next session?
Did the patient meet the intended cardiovascular intensity for the session?	Do you have a plan developed in how to progress this patient for the next session or coming weeks?	How will you modify the device settings to appropriately adjust challenge (increase or decrease) based on this session?

Formal outcome reassessment should be performed, at minimum, upon initial evaluation and at discharge, but ideally more frequently between these intervals depending on the practice setting [32]. Honest acceptance of failures, and celebration of success with exoskeleton use is essential to developing expertise and effective exoskeleton implementation in a clinical setting. Each session where technology is thoughtfully applied can provide insight towards broadening the application of its use with future patients presenting in similar patterns or diagnoses. Furthermore, this could result in transference to other patient populations with similar impairments but have been yet to be investigated.

## Framework utilization

As the authors of this manuscript and practicing clinicians, we have applied this framework across multiple levels of rehabilitation settings, from inpatient to outpatient with varying levels of chronicity. Use of this framework provides a systematic approach to considering which patients may be appropriate for exoskeleton utilization and determining which device may be most beneficial based on a patient’s impairments and presentation. Through attempted implementation and reflection, this framework has also helped us to identify when exoskeletons are not appropriate, whether with the initiation of care, or after a brief usage trial. We feel the use of this framework within our organization assisted us with a decrease in the therapist perception of technology burden and barriers and improved appropriate utilization patterns.

## Conclusion

Though the current exoskeleton body of literature is limited, the quality and quantity of clinical trials investigating these technologies continue to improve. This manuscript proposes a structured approach for clinicians to consider when exoskeletons may benefit their patients and thoughtfully choose an available exoskeleton to suit their patient’s goals of improving ambulatory function. Exoskeletons are costly investments, require clinician training, are time intensive and have a potential for insurance reimbursement issues. Despite these limitations, it should not deter the advanced facility from making the investment and encouraging the translation of current best practice along with informed decision making with frequent reflection of the effect utilization on patient outcomes. Future research is needed to aid clinical application through better defined protocols, and improved use of objective clinical outcomes measures sensitive to change. In addition, exoskeleton research should focus on dosage, intensity and frequency parameters which apply principles known to promote neurological recovery and are more applicable to the constraints of the clinical environment.

## Data Availability

Data sharing is not applicable to this article as no datasets were generated or analyzed during the current study.

## Abbreviations

FDA: Food & Drug Administration.
HAL: Hybrid Assistive Limb.
WAD: Walking Assist Device.
B LE: bilateral lower extremity.
U LE: unilateral lower extremity.
FP: fully powered.
PA: partially assistive.
SSV: Self-Selected Velocity.
FV: Fast Velocity.
2MWT: 2min walk test.
SCI: spinal cord injury.
CPG: clinical practice guideline.
AFO: ankle foot orthosis.
RPE: rating of perceived exertion.
PT: physical therapy.
OT: occupational therapy.
SLP: speech therapy.
